# 

**Published:** 1908-06-13

**Authors:** 


### BOOKS RECEIVED.

H. Renshaw.

"A Practice of Medicine." 2 vols. By Sir William Whitla, M.A., M.D.

C. Griffin and Co., Ltd.

"Infancy and Infant Rearing." By J. B. Hellier, M.D. 2nd edition.

Henry Frowde and Hodder and Stoughton.

"Diseases of the Spinal Cord." By R. T. Williamson, M.D.

J. B. Lippincott Co.

"International Clinics." 18th Series. Vol. I.

### THE GRADUATES' MEDICAL SCHOOLS.

DIARY FOR WEEK JUNE 15 TO 20.

**ROYAL COLLEGE OF PHYSICIANS, Pall Mall East.**
Croonian Lectures.

**At 5 p.m.**
**June 18,** Dr. A. E. Garrod, **Inborn Errors in Metabolism.**

**THE HOSPITAL FOR SICK CHILDREN, Great Ormond Street, W.C.**

**At 4 p.m.**
**June 18,** Mr. Fairbank, **The "Acute Abdomen" in Children.**

**NATIONAL HOSPITAL FOR THE PARALYSED AND EPILEPTIC, Queen Square, Bloomsbury.**

**At 3.30 p.m.**
**June 16,** Dr. Collier, **Amyotonia Congenita.**
**June 19,** Dr. Collier, **Caisson Disease.**

**MOUNT VERNON HOSPITAL, Hampstead.**

**At 5 p.m.**
**June 18,** Mr. Harold Barwell, **Cases of Laryngeal Disease.**

**CHARING CROSS HOSPITAL, W.C.**

**At 4 p.m.**
**June 18,** Dr. Jewesbury, **Medical.**

**THE THROAT HOSPITAL, Golden Square, W.**

**At 5.30 p.m.**
**June 15,** Dr. H. W. F. Powell, **Inflammatory Affections of the Naso-Pharynx and Pharyngeal Tonsils.**
**June 18,** Dr. J. W. Bond, **Acute Inflammatory Affections of the Oropharynx and Tonsils.**

**NORTH-EAST LONDON POST-GRADUATE COLLEGE, Prince of Wales's General Hospital, Tottenham, N.**

**At 4.30 p.m.**
**June 17,** Dr. J. E. Squire, C.B., **Demonstration of Cases at the Northwood Sanatorium.**

**MEDICAL GRADUATES' COLLEGE AND POLYCLINIC, Chenies Street, W.C.**

**At 5.15 p.m.**
**June 15,** Professor Hewlett, **The Diagnosis of Syphilis by the Antigen Method.**
**June 16,** Mr. J. Howell Evans, **Injuries to the Head of the New Born.**
**June 17.** Mr. C. B. Keetley, **Affections of the Toes, Feet, and Ankles.**
**June 18,** Dr. W. Langdon Brown, **Diseases Associated with Cyanosis.**

**LONDON SCHOOL OF CLINICAL MEDICINE, Seamen's Hospital, Greenwich, S.E.**

**At 2.15 p.m.**
**June 19,** Dr. Rose Bradford, **Peripheral Neuritis.**

**WEST LONDON HOSPITAL, Hammersmith Road.**

**At 12 noon.**
**June 15,** Dr. Low, **Pathological Demonstration.**
**At 12.15 p.m.**
**June 17 and 19,** Dr. Pritchard, **Practical Medicine.**
**At 5 p.m.**
**June 15,** Mr. Bidwell, **Plaster of Paris Splints—I.**
**June 16,** Mr. Armour, **Lymphatic Gland Enlargements.**
**June 17,** Mr. Armour, **Thyroid Enlargements.**
**June 18,** Mr. Baldwin, **Practical Surgery—IV.**
**June 19,** Dr. R. H. Cole, **Medico-Legal Relations of Insanity.**

**LONDON HOSPITAL MEDICAL COLLEGE, Turner Street, Mile End, E.**

**At 3 p.m.**
**June 16,** Mr. Jonathan Hutchinson, F.R.S., **The Liver and Jaundice in reference to Skin Diseases.**

**ROYAL INSTITUTE OF PUBLIC HEALTH, 37 Russell Square, W.C.**
Harben Lectures.

**At 5 p.m.**

**June 17,** Professor G. H. F. Nuttall, **Spirochœtosis in Man and Animals.**

THE HOSPITAL *June 13, 1908.*

*Name* ..................................................

*Address* ..................................................

..................................................

This Coupon must accompany manuscript or contributions intended for THE HOSPITAL.

